# Research on Automatic Recognition of Dairy Cow Daily Behaviors Based on Deep Learning

**DOI:** 10.3390/ani14030458

**Published:** 2024-01-30

**Authors:** Rongchuan Yu, Xiaoli Wei, Yan Liu, Fan Yang, Weizheng Shen, Zhixin Gu

**Affiliations:** 1 College of Computer and Control Engineering, Northeast Forestry University, Harbin 150040, China; 2 College of Electric and Information, Northeast Agricultural University, Harbin 150030, China

**Keywords:** YOLO, dairy cow, behavior detection, dense module, multi-scale detection heads

## Abstract

**Simple Summary:**

Dairy cow behavior detection is of great significance for cattle health management. Through the detection of the four behaviors of dairy cows—standing, lying, eating, and drinking—we can gain valuable insights into the well-being of cows. For example, hoof disease can increase the amount of time a cow lies down, and digestive system issues can cause a decrease in food intake. Visual inspection of cow behavior can keep track of changes in cow behavior, and non-invasive detection can reduce cow discomfort and improve animal welfare. In this study, we employed computer vision-based deep learning techniques for the detection of cow behavior, and experimental results demonstrated its promising application in real farm settings.

**Abstract:**

Dairy cow behavior carries important health information. Timely and accurate detection of behaviors such as drinking, feeding, lying, and standing is meaningful for monitoring individual cows and herd management. In this study, a model called Res-DenseYOLO is proposed for accurately detecting the individual behavior of dairy cows living in cowsheds. Specifically, a dense module was integrated into the backbone network of YOLOv5 to strengthen feature extraction for actual cowshed environments. A CoordAtt attention mechanism and SioU loss function were added to enhance feature learning and training convergence. Multi-scale detection heads were designed to improve small target detection. The model was trained and tested on 5516 images collected from monitoring videos of a dairy cowshed. The experimental results showed that the performance of Res-DenseYOLO proposed in this paper is better than that of Fast-RCNN, SSD, YOLOv4, YOLOv7, and other detection models in terms of precision, recall, and mAP metrics. Specifically, Res-DenseYOLO achieved 94.7% precision, 91.2% recall, and 96.3% mAP, outperforming the baseline YOLOv5 model by 0.7%, 4.2%, and 3.7%, respectively. This research developed a useful solution for real-time and accurate detection of dairy cow behaviors with video monitoring only, providing valuable behavioral data for animal welfare and production management.

## 1. Introduction

The real-time and precise monitoring of dairy cows’ daily behaviors is crucial in large-scale intensive farming systems, as animal behaviors are closely correlated with their health and welfare conditions [1]. Changes in behaviors such as drinking, feeding, lying, and standing can serve as early warnings and indicators of disease occurrence. Cows afflicted with foot rot may display signs of lameness, reduced locomotion, or increased resting time [2]. Similarly, cows suffering from mastitis are likely to experience decreased feed intake and spend less time feeding [3]. An increase of considerable magnitude in the duration of cow resting periods often indicates an elevated probability of disease, which exhibits an inverse correlation with milk production [4]. In recent years, manual observation has remained the predominant approach for behaviors; however, it is characterized by inefficiency, time consumption, and susceptibility to subjective biases introduced by human observers [5].

Currently, dairy cow behavior detection methods are primarily categorized into two approaches: contact-based detection and non-contact image recognition detection. Contact sensors typically require animals to wear them in specific positions to collect motion and physiological data for identifying targeted animal behaviors. Arcidiacono et al. [6] attached acceleration sensors to cows’ hind legs and proposed a threshold-based acceleration algorithm for detecting estrus behaviors. Schweinzer et al. [7] used a 3D acceleration sensor system based on ear tags to collect cow behavior data and detect behaviors during estrus and pregnancy.

However, contact sensors may elicit discomfort or resistance in animals, thereby impeding accurate detection due to potential interference from external noise [2]. Moreover, the reliance on contact sensors necessitates periodic battery replacement and increases susceptibility to loss, thus imposing limitations on long-term monitoring [8].

In recent years, the application of non-contact image recognition in animal behavior identification has gained significant traction. Meunier et al. [8] used integrated graphic analysis techniques to extract dairy cow behavior features and classify behaviors such as standing and lying. Wang et al. [9] utilized Siamese networks to extract features from before and after feeding-trough images of dairy cows. By analyzing the differences between the two features, they obtained data on dairy cow feed intake, achieving non-contact measurement of feed intake in dairy cows. Shi et al. [10] proposed a cow body condition score automatic estimation method based on attention-guided 3D point cloud feature extraction and established a 3D data format dataset for estimating cow body condition score, enabling a better assessment of cow physical condition. However, traditional machine learning still necessitates manual feature extraction, lacks scalability, and may not perform well on high-dimensional data, thereby presenting challenges for its application in large-scale intensive farming environments.

With the continuous development and progress of deep learning, more applications have emerged in animal behavior detection [11]. Gao et al. [12] proposed a hybrid model that combines a Convolutional Neural Network (CNN) and a Gated Recurrent Unit (GRU) and designed and integrated a specific spatiotemporal attention mechanism in the CNN–GRU hybrid model to identify aggressive behaviors of group-housed pigs automatically and accurately. Zheng et al. [13] used the Faster R-CNN image classification algorithm to recognize behaviors such as standing, sitting, lying, and lateral lying of pigs in farming. Yin et al. [14] used a deep learning method based on EfficientNet and long short-term memory architecture for rapid recognition of dairy cows’ daily behavior data. Li et al. [15] achieved automatic detection of scratches on metallic sliding components using WearNet. Compared to other lightweight CNN models, WearNet had the advantages of smaller model size and faster detection speed. However, traditional deep learning applications require algorithms to first generate sample candidate boxes and then classify samples through convolutional neural networks, which cannot meet the fast real-time detection requirements.

The YOLO algorithm is a one-stage fast object detection algorithm. Compared with other region-based CNN detection models, the YOLO model uses a single CNN network to simultaneously complete target detection and classification tasks, thereby integrating the entire process as a regression problem. Consequently, YOLO exhibits advantages in real-time detection scenarios [16]. Hu et al. [17] employed the YOLO algorithm to localize cows in videos and utilized a segmentation algorithm to extract images of the cow’s head, trunk, and legs. Then, feature extraction and fusion were performed on these three parts to achieve cow identification. Wang et al. [18] optimized anchor box sizes and boundary box loss functions based on the YOLOv3 model to quickly identify estrus behaviors in dairy cows. Kawagoe et al. [19] used a YOLO detector to capture cow heads eating from videos and applied transfer learning for detection of cow feeding time. Guo et al. [20] used a YOLOv3-tiny model for cow individual identification and used eye temperature recognition technology to measure cow body temperature, realizing non-invasive identification of cow temperature and individuals. In summary, although YOLO achieves real-time detection, further improvement is required for its accuracy in dairy cow behavior detection, and it is susceptible to the influence of actual cowshed environments.

The aim of this study was to improve the algorithm on the basis of YOLOv5, enabling the model to precisely identify diverse behaviors exhibited by cows in actual dairy cowshed environments. First, in actual farming environments, cow behavior identification is easily affected by natural light, object occlusion, and cow clustering. Background masking was applied to the original data to remove environmental noise interference. Secondly, the Res-Dense module, designed as the backbone network in this study, replaced the original YOLOv5’s CSPDarkNet53 backbone network to enhance the network’s capacity for extracting image features. The CoordAtt attention mechanism and four detection heads were integrated into the original backbone network to strengthen the ability to extract image features and improve the accuracy of small target detection. SioU regression loss function was introduced to accelerate network convergence. Finally, ablation experiments and performance comparisons with other target detection algorithms were conducted to validate that the proposed model achieves better detection effects for dairy cow behaviors in actual environments.

## 2. Materials and Methods

### 2.1. Data Sources

This study was conducted at Shengkang Livestock Cowshed located in Daqing City, Heilongjiang Province, China. The cowshed housed 900 Holstein dairy cows. A total of 90 healthy cows were included in this experiment. The cows were fed twice a day, from 5:00 to 6:00 in the morning and from 17:00 to 18:00 in the afternoon. Therefore, video clips of the cows’ eating behaviors were collected during these two periods. The cowshed’s surveillance cameras switched to infrared shooting mode at 20:00 daily. Clips of cows’ lying behavior were cut from the surveillance video from 20:00 to 3:00 am the next day. Cow standing and drinking behaviors were cut from the surveillance video during daylight hours when there was sufficient light. Video data were recorded between 1 July 2022 and 1 September 2022.

**Figure 1:**
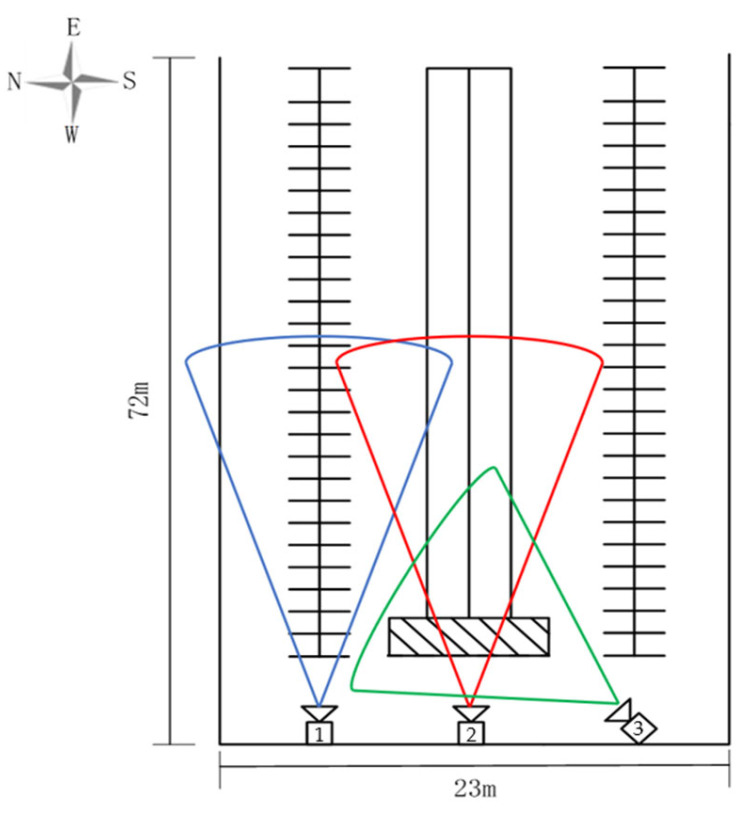
Schematic diagram of the cowshed. The blue area captured by camera 1 is responsible for capturing cows’ standing and lying behavior; the red area captured by camera 2 is responsible for capturing cows’ eating behavior; and the green area captured by camera 3 is responsible for capturing cows’ drinking behavior.

Three video collection points were selected in the experiment, as shown in Figure 1. We selected three surveillance cameras with 4 million pixels (Hangzhou Hikvision Digital Technology Co., Ltd., Hangzhou, China, DS-IPC-B14H-LFT), capturing images of the cows’ behaviors. The height of these three cameras from the ground was three meters. Among these, two were 15 degrees from the ground to record the behaviors of cows in terms of their standing, lying, and eating behaviors. The third one was at an angle of 35 degrees to the ground to record drinking behaviors. Finally, 35 video clips were extracted from the recorded videos, each lasting 2 min. The clips covered cow behaviors such as standing, feeding, drinking, and lying, both during daytime with color images and during nighttime with black-and-white infrared images. Python scripts were used to extract one screenshot every 12 frames from the videos. Repeated and out-of-focus images were removed, resulting in a total of 5516 images.

**Figure 2:**
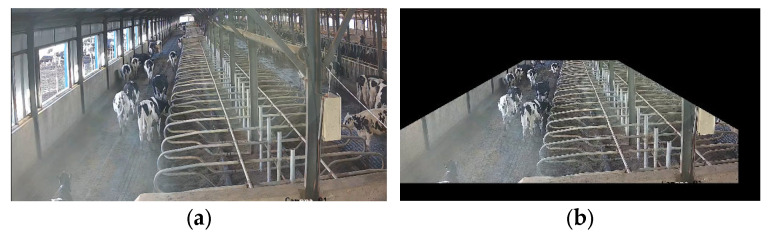
Image masking processing: (**a**) original image of standing cow; (**b**) image after black masking of unrelated areas.

When we observed the collected dairy cow behavior videos, we found that some areas captured by the cameras interfered with the experiment. For example, direct sunlight illumination caused overexposure in the edge areas of the cowshed, as shown in Figure 2a. These areas unrelated to the experiment would increase the model computation and error recognition rate during cow behavior detection. Cow behaviors were usually associated with fixed locations. For example, drinking and feeding behaviors always occurred at locations of water troughs and feed troughs, while standing behaviors were predominantly observed in passage areas. Therefore, an image masking algorithm was employed to obscure outdoor regions and areas unrelated to standing behaviors using black masks, as shown in Figure 2b, without impeding normal behavioral patterns.

### 2.2. YOLOv5

**Figure 3:**
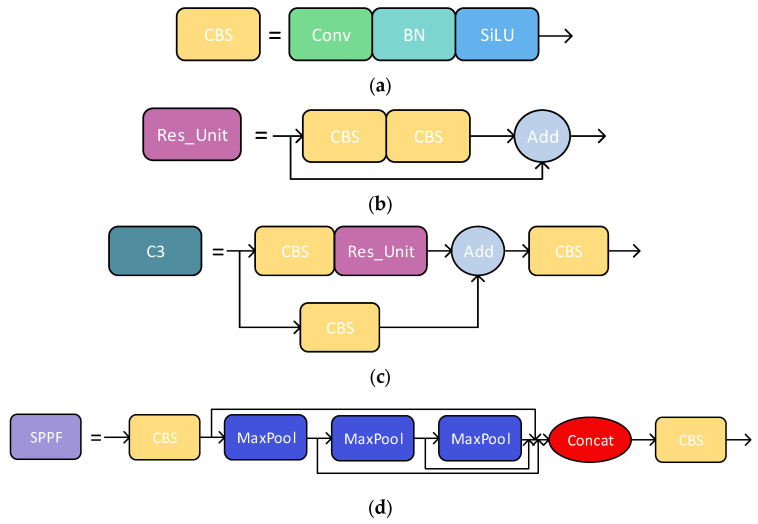
YOLOv5 basic module diagram: (**a**) CBS block; (**b**) Res_Unit; (**c**) C3 block; (**d**) SPPF structure.

The YOLOv5 algorithm, proposed by Ultralytics LLC (Los Angeles, CA, USA,) in May 2020, is a one-stage tar-get detection method that employs a single CNN module to achieve end-to-end target detection. Compared with YOLOv4 or earlier versions, YOLOv5 has faster speed and higher accuracy. It consists of three parts: backbone, neck, and head. CSPDarkNet53 is used as the feature extraction backbone network. Path Aggregation Network is used as the bottleneck structure. The output after convolution is finally provided to the detection head for image classification and boundary box regression, as shown in Figure 3.

The backbone network CSPDarkNet53 mainly consists of CBS, Res_Unit, C3, and SPPF modules. The CBS module encompasses convolutional operations, batch normalization, and SiLu activation function (Figure 3a). The Res_Unit module is a classic residual structure that combines two pieces of information by summation and continues to transmit downward (Figure 3b). Within this structure, the input image is divided into two paths: one path performs bottleneck operations to reduce the dimensions of the image, while the other path continuously applies bottlenecks based on the network structure to ensure consistent input and output image sizes.

Finally, the outputs of all layers are concatenated and undergo 1 × 1 convolution to reduce the number of output channels to obtain the final output. The C3 module is the core module of this network. Its input is divided into two branches. The first branch halves the number of feature map channels after the CBS module, while the second branch concatenates the feature map of the first branch after the CBS module and multiple Bottleneck modules. Finally, a CBS module outputs the feature map (Figure 3c). SPPF is in the last layer of the backbone (Figure 3d). It connects the outputs of three pooling layers through Concat after changing the feature dimensions with a CBS module. Each pooling feature will become the input of the next pooling, achieving faster speed than SPP.

### 2.3. Model Improvement

#### 2.3.1. DenseNet Module

**Figure 4:**
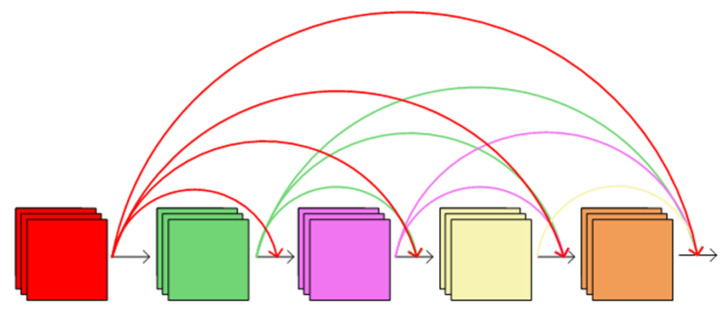
DenseNet model diagram.

As the convolutional modules are stacked, the feature information gradually diminishes during the convolution and downsampling process. Due to the complexity of the cowshed environment, the loss of feature information increased the difficulty of recognition and reduced recognition accuracy. DenseNet [21] can significantly improve the efficiency of feature information utilization. It achieves this by densely connecting each layer with all preceding layers in a feed-forward manner while preserving the same number of channels, as shown in Figure 4. The core expression of DenseNet is as follows:

(1) $$X_{t}=H_{t}(\left[X_{0},X_{1},\ldots ,X_{t-1}\right])$$

In the equation, $X_{t}$ represents the output of the t-th layer of the network. $\left[X_{0},X_{1},\ldots ,X_{t-1}\right]$ represents the feature maps from the 0th layer to the (t − 1)th layer, which are fused together using the DenseNet module. $H_{t}$ represents the combination of batch normalization, rectified linear units’ activation, and convolutional operations.

#### 2.3.2. CoordAtt Attention Mechanism

Considering the actual environment of the cowshed and aiming to enhance the model’s ability to represent different behaviors of cows, this study introduced four attention mechanism modules preceding the four detection heads of YOLOv5. Attention mechanisms allocated weights to focus the network’s attention on relevant regions or features associated with the current task, thereby selectively attending to and processing specific fragments of information to improve neural networks’ performance.

**Figure 5:**
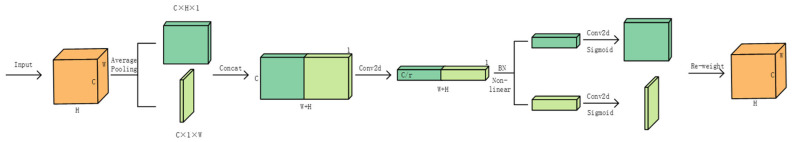
CoordAtt attention mechanism.

Attention mechanisms can generally be divided into two major categories: spatial attention and channel attention. Traditional attention mechanisms such as SE [22] and CBAM [23] have been well applied in various domains. However, the SE attention module tends to focus more on inter-channel information while neglecting positional information. CBAM attempts to extract positional attention information through convolution after reducing the number of channels, but convolution can only capture local relationships and lacks the ability to extract long-range relationships. Therefore, this paper adopts the CoordAtt attention mechanism [24], as shown in Figure 5, which can encode both horizontal and vertical positional information into channel attention. This enables the network to obtain a wide range of positional information without introducing excessive computational complexity, thereby reducing computation while improving model robustness.

When the feature maps are input into the CoordAtt module, the channel attention is divided into two parallel one-dimensional feature encodings, which aggregate features along the horizontal and vertical directions, respectively. The feature maps are then convolved and activated with an activation function. This allows capturing long-range dependencies along one spatial direction while preserving accurate positional information along the other spatial direction. The resulting feature maps, composed of horizontal and vertical directions, will focus on the parts we need to pay attention to.

#### 2.3.3. Four Prediction Head Structure

When analyzing the dataset of cow images, it was observed that due to the rectangular structure of the cowshed, when cows were positioned at the end of the camera’s field of view, their behavior images often occupied a smaller proportion of the entire image. After multiple layers of feature extraction, the small-scale cow behavior targets could lose some of their feature information, ultimately affecting the accuracy of cow behavior recognition.

**Figure 6:**
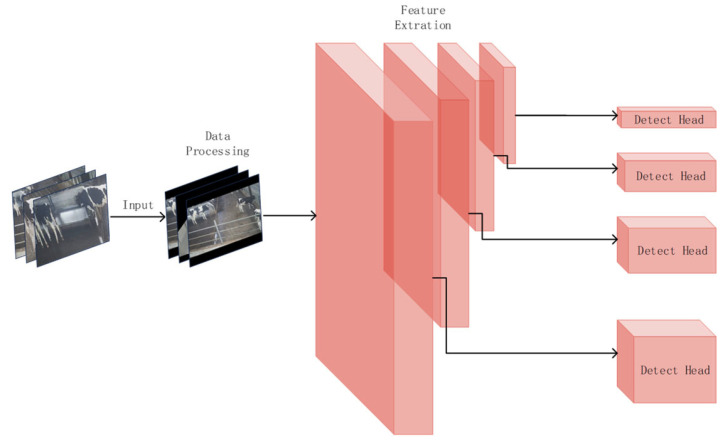
Four-prediction-head structure.

To address the issue of detection of small objects, an additional prediction head for detecting small objects was added to the existing three object detection heads in YOLOv5. The new four-prediction-head structure helped alleviate the negative impact caused by drastic variations in target scales. The enhanced network model could extract more comprehensive features from the underlying network and showed improvements in issues such as false positives, false negatives, and low confidence. The overall structure of the model’s prediction heads is illustrated in Figure 6.

#### 2.3.4. SioU Loss

In the early stages of object detection, IoU [25] was one of the most used evaluation metrics. It measures the overlap between predicted bounding boxes and ground truth boxes to assess the algorithm’s detection performance. GioU [26] considers the distance between the center points and aspect ratios of predicted and ground truth boxes while retaining the advantages of IoU. Subsequently, DioU [27] and CioU [28] were proposed. DioU improves the relative position optimization between predicted and ground truth boxes, while CioU introduces a scale factor to balance the differences in aspect ratios. Although these loss functions have corresponding improvements, they fail to address the issue of mismatched orientations between predicted and ground truth boxes, resulting in slow convergence and low efficiency of the entire model. Predicted boxes may move in random directions during training, leading to poorer models. To address this problem, we introduced the SioU [29] regression loss function. This loss function considers the vector angle between the desired regressions and redefines the penalty metric. This approach allows the predicted boxes to quickly move towards the nearest axis, and subsequent methods only require regression of a single coordinate, either X or Y.

The SIoU regression loss function consists of four components: angle loss, distance loss, shape loss, and IoU loss. The angle loss is defined as follows:

(2) $$ᐱ=1\;-\;2\times \sin^{2}\left(\arcsin\left(\frac{c_{h}}{\sigma }\right)\;-\;\frac{\pi }{4}\right)$$

(3) $$\sigma =\sqrt{{\left(b_{c_{x}}^{\mathrm{gt}}\;-\;b_{c_{x}}\right)}^{2}+{\left(b_{c_{y}}^{\mathrm{gt}}\;-\;b_{c_{y}}\right)}^{2}}$$

(4) $$\;\;\;c_{h}=\max\left(b_{c_{y}}^{\mathrm{gt}},b_{c_{y}}\right)\;-\;\min\left(b_{c_{y}}^{\mathrm{gt}},b_{c_{y}}\right)$$

**Figure 7:**
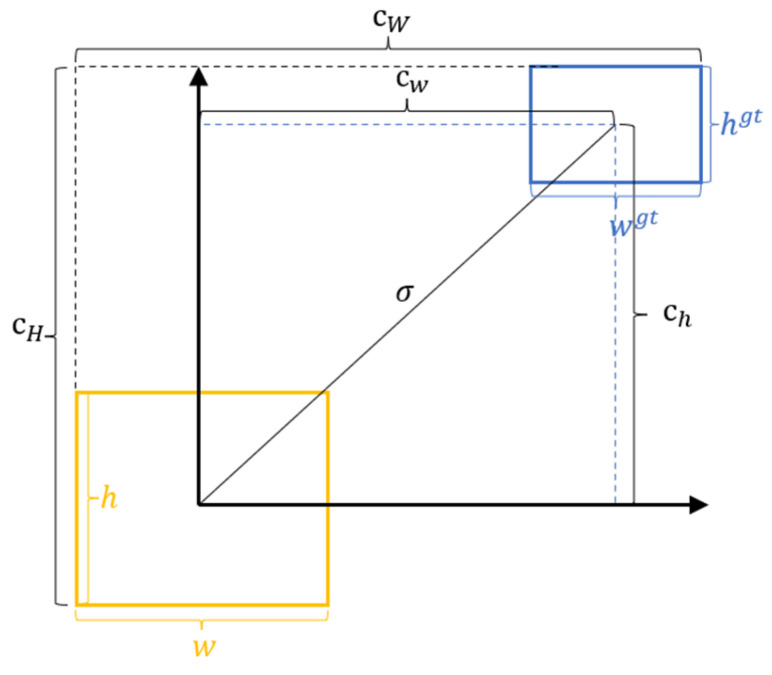
SioU illustration. The yellow box represents the predicted bounding box, and the blue box represents the actual target box.

In the SioU regression loss function, $c_{h}$ represents the height difference between the center points in the actual coordinate system and the predicted coordinate system, while σ represents the distance between the center points in the actual coordinate system and the predicted coordinate system. $b_{c_{x}}^{\mathrm{gt}}\;$ and $\;b_{c_{y}}^{\mathrm{gt}}\;$ denote the center coordinates of the actual object to be detected, while $b_{c_{x}}\;\mathrm{and}\;b_{c_{y}}$ represent the center coordinates of the predicted bounding box generated by the model, as illustrated in Figure 7.

The distance loss is defined as follows:

(5) $$\Delta =2\;-\;e^{-\gamma \rho_{x}}\;-\;e^{-\gamma \rho_{y}}$$

(6) $$\begin{array}{c} \rho_{x}={\left(\frac{b_{c_{x}}^{\mathrm{gt}}\;-\;b_{c_{x}}}{c_{W}}\right)}^{2},\rho_{y}={\left(\frac{b_{c_{y}}^{\mathrm{gt}}\;-\;b_{c_{y}}}{c_{h}}\right)}^{2} \end{array}$$

(7) γ=2 −ᐱ

$c_{w}$ and $c_{h}\;$ represent the widths and heights of the minimum enclosing rectangles of the actual bounding box and the predicted bounding box, respectively.

The shape loss is defined as follows:

(8) $$\Omega ={\left(1\;-\;e^{-\omega_{w}}\right)}^{\theta }+{\left(1\;-\;e^{-\omega_{h}}\right)}^{\theta }$$

(9) $$\;\;\omega_{w}=\frac{\left|w\;-\;w^{\mathrm{gt}}\right|}{\max\left(w,w^{\mathrm{gt}}\right)}{,\omega }_{w}=\frac{\left|h\;-\;h^{\mathrm{gt}}\right|}{\max\left(h,h^{\mathrm{gt}}\right)}$$

w, h, $\;w^{\mathrm{gt}}$, and $h^{\mathrm{gt}}\;$ represent the widths and heights of the predicted bounding box and actual target box, respectively. θ is an adjustable variable that represents the weight assigned by the network to the shape loss.

The SioU loss function is defined as follows:

(10) $$\;\mathrm{Loss}=1\;-\;\mathrm{IoU}+\frac{\Delta +\Omega }{2}$$

(11) $$\;\mathrm{IoU}=\frac{A\cap B}{A\cup B}$$

### 2.4. Res-DenseYOLO Model

**Figure 8:**
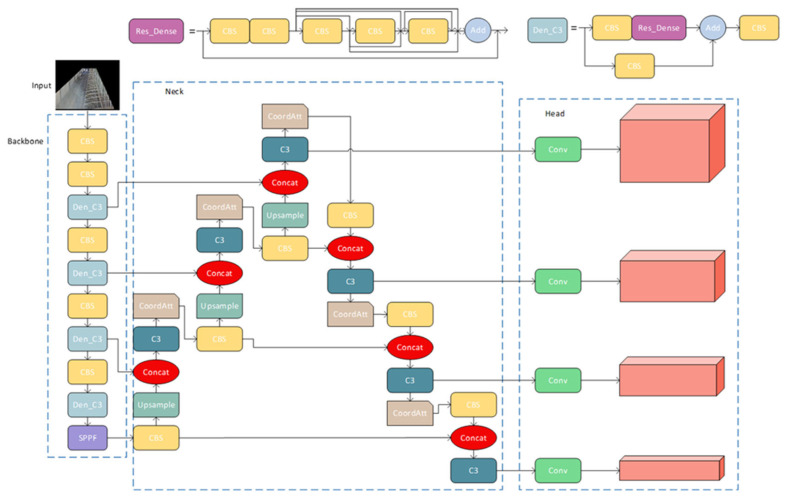
The improved Res-DenseYOLO model.

After the aforementioned model optimization, the enhanced YOLOv5 network is illustrated in Figure 8. In the backbone layer responsible for feature extraction, we replaced the original C3 module with the Den_C3 module. When the feature information was propagated from the upper layers to the Den_C3 module, it was split into two paths for further transmission. One path directly transmitted the information to the next layer through a CBS module. The other path first passed through a 1 × 1 CBS module to reduce the number of channels and then undergoes feature fusion in the Res-Dense module. Referring to the design idea of DenseNet, we encapsulated five CBS modules into the Res_Dense module. Since the feature map extracted from the first CBS module was shallow, we transferred its feature data directly to the next module. The feature maps from the second CBS module would be fused with the features from each subsequent layer. This greatly improved the feature extraction capability. The two paths were merged through Concat for information fusion, and finally, the feature information was extracted again through a CBS module and input into the subsequent network modules.

On the one hand, the feature information was preserved intact by utilizing the residual network structure, where it was directly input to the next layer through a CBS module. This helped maintain the integrity of the feature information. On the other hand, the dense connection network module enriched the network’s feature information and avoided the loss of feature information that may occur as the network deepens. Therefore, compared to the original YOLOv5 backbone network, the improved backbone network had a more abundant and accurate feature extraction capability.

In the neck layer, we incorporated the CoordAtt attention mechanism to allow the network to focus more on valuable feature extraction parts. Since the Den_C3 module increased the number of parameters in the network, while the neck layer was responsible for multi-scale feature fusion on the feature maps, we could retain the original C3 module to handle this task. The initial input size of the network was 640 × 640, and after undergoing upsampling and downsampling operations in the network, the detection heads in the head layer were divided into four different sizes. Compared to the original model, the improved detection heads had a minimum size of 40 × 40, which significantly enhanced the detection of cows at the camera edges and cows at the far end of the lens.

## 3. Results and Discussion

### 3.1. Experiment Environment

The computer configuration used for the experiments is listed in Table 1.

During the model training process, the performance of the model was strongly correlated with certain hyperparameter settings. Additionally, different datasets may require adjustments to the hyperparameters. For example, the batch size determines the number of images in each training batch. Increasing the batch size can shorten the training time, but if the batch size is increased from 16 to 24, it may exceed the memory capacity of the GPU, resulting in training failure. The learning rate is a hyperparameter that controls the speed of weight updates in the model. If the learning rate is too large, the model may miss the optimal solution and experience oscillation or fail to converge. On the other hand, if the learning rate is too small, the convergence speed of the model may slow down, or it may get stuck in a local optimum. Therefore, selecting appropriate hyperparameters plays a crucial role in model performance, as shown in Table 2.

### 3.2. Evaluation Metrics

To evaluate the performance of the proposed cow behavior recognition model, we chose precision, recall, and mean average precision (mAP) as the evaluation metrics. The formulas defining these metrics are as follows:

(12) $$\;\mathrm{Precision}=\frac{\mathrm{TP}}{\mathrm{TP}+\mathrm{FP}}$$

(13) $$\;\mathrm{Recall}=\frac{\mathrm{TP}}{\mathrm{TP}+\mathrm{FN}}$$

(14) $$\;\mathrm{AP}=\int_{0}^{1}p\left(r\right)\mathrm{dr}$$

(15) $$\;\mathrm{mAP}=\frac{\sum_{i=1}^{k}AP_{i}}{k}$$

When the intersection between the predicted frame and the actual annotated frame was greater than a specified threshold, the predicted frame was labeled as a positive sample. Otherwise, it was labeled as a negative sample. True Positive (TP) represents the number of correctly classified positive samples, False Positive (FP) represents the number of falsely classified positive samples, and False Negative (FN) represents the number of falsely classified negative samples. AP refers to the area under the precision-recall curve of a detection model for a specific category. mAP, on the other hand, is the mean Average Precision obtained by averaging the AP values across multiple categories. The parameter k represents the number of classes.

### 3.3. Experimental Results

#### 3.3.1. Ablation Experiment

To validate the effectiveness of the proposed improvement method in this study, we conducted comparative experiments on the backbone network, loss function, and attention mechanism. All experiments were conducted using YOLOv5 as the baseline model. As the primary aim of this study was to enhance the accuracy of cow behavior recognition, we opted for precision, recall, and mAP as the evaluation metrics to measure the model’s performance. High accuracy means that the algorithm identified a high percentage of correct cow behaviors, which means that our model reduced the number of incorrect predictions and thus reduced the potential losses due to incorrect predictions. For instance, misjudgments of cows’ behaviors can result in unnecessary or delayed treatments. On the other hand, having a high recall rate is equally crucial, as it guaranteed that most of the cows’ behaviors had been correctly identified, ensuring that we did not overlook any important behavioral indicators. Specifically, for early disease detection in cows, a model delivering high recall indicates that we can notice any abnormal behaviors sufficiently early, allowing us to take immediate appropriate measures to ensure the well-being of the cows.

Firstly, to compare the performance of the Res-Dense module as the backbone network, we replaced the YOLOv5 backbone with ShuffleNetV2 and EfficientNet. From the results in Table 3, it can be observed that compared to other models with different backbone networks, the Res-Dense module improved the precision by 0.7%, and the recall and mAP also increased by 0.3% and 0.4%, respectively. These metrics validate the performance of the Res-Dense module in accurately recognizing cow behaviors in actual cowshed environments.

Next, a comparison was conducted on the Res-Dense module, evaluating various commonly utilized loss functions such as GioU, CioU, and DioU against the SioU loss function chosen for this study. The outcomes of this analysis are presented in Table 4.

From Table 4, it can be observed that compared to the underperforming DioU, the SioU loss function improved the precision and recall by 0.4% and 2.1%, respectively. The initial YOLOv5 model selected CioU as the loss function, which demonstrated some improvement over DioU and GioU in terms of performance. However, when using the SioU loss function, there was an improvement of 0.3% in precision, 1.2% in recall, and 0.2% in mAP.

The experimental results indicated that the addition of angle penalty cost in the loss function, combined with the Res-Dense module, performed well in cow behavior recognition and detection tasks. The SioU loss function, with its integration of angle penalty, contributed to higher precision, recall, and mAP values, enhancing the model’s accuracy in identifying and classifying cow behaviors.

To evaluate the performance of different attention mechanisms in the current network, this study incorporated the SE, CBAM, and CoordAtt mechanisms into the backbone network with the Res-Dense module for training and comparison. The experimental results are presented in Table 5.

The SE attention mechanism ignored positional information and performed poorly among the three attention mechanisms. On the other hand, CBAM considered both channel and positional information, enhancing the feature representation capability of the feature maps. Thus, adding the CBAM attention mechanism to the backbone network improved the network performance, although the effect was not significant. The CoordAtt attention mechanism embedded positional information into channel attention by capturing long-range relationships in one direction while preserving spatial information in another direction. Referring to the literature by Zheng et al. [30], our findings were consistent with their conclusion that after incorporating CoordAtt into the backbone network, compared to CBAM, there was an improvement of 1% in precision, 0.3% in recall, and 1.2% in mAP.

Through ablation experiments, the positive impact of the proposed improvement strategies on the initial YOLOv5 network was validated. The performance improvement varied when different improvement strategies were added to the network model. From the data in Table 6, we can see that when only the Res-Dense module was added to the original YOLOv5 model, the accuracy of the network decreased by 1.6%, recall decreased by 1.3%, and mAP decreased by 1.3%. After introducing the CoordAtt attention mechanism, the model achieved a 0.4% improvement in precision, 0.7% improvement in recall, and 0.7% improvement in mAP. It can be observed that the introduction of the CoordAtt attention mechanism enhances the model’s ability to analyze features in both channel and spatial dimensions, leading to further performance improvement. When the SioU module was added, the performance improvement was relatively small, with only a 0.2% improvement in precision and 0.3% improvement in mAP. Finally, when the multi-head detection module was added, compared to the model without the additional detection head, there was a 1% improvement in precision, 0.7% improvement in recall, and 0.3% improvement in mAP, demonstrating the effectiveness of introducing the multi-head detection module for performance improvement.

In order to validate the effectiveness of the improved object detection algorithm in practical cow behavior detection and further analyze the performance of the algorithm itself, we selected several classic object detection models, including SSD, Faster RCNN, YOLOv4, original YOLOv5, and YOLOv7. We compared them comprehensively with the improved Res-DenseYOLO model using precision, recall, and mAP as the performance evaluation metrics, as shown in Table 7.

From the data in the table, it can be observed that in our collected cow behavior dataset, the Res-DenseYOLO model outperforms other models in terms of accuracy, recall, and mAP. Among the two-stage detection models, SSD exhibited higher performance. However, the Res-DenseYOLO model showed improvements of 2.3% in precision, 2.9% in recall, and 5.5% in mAP, and it is a single-stage detection model, which also has advantages in detection speed.

Among the YOLO series algorithms, we selected the relatively newer YOLOv7-tiny model as a comparison. The experimental results showed that within the initial YOLO detection series, the YOLOv7-tiny model performed better than other YOLO models. However, compared to the Res-DenseYOLO model, there was still a gap of 0.8% in precision, 2.2% in recall, and 2.1% in mAP. Therefore, in cow behavior detection, the Res-DenseYOLO model can meet the requirements of faster and more accurate cow behavior detection.

#### 3.3.2. Feature Heatmap Analysis

**Figure 9:**
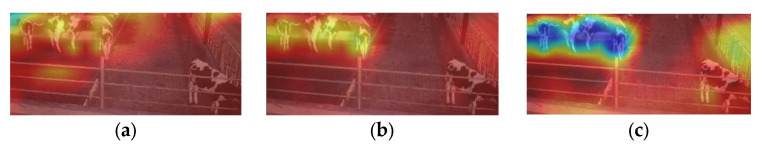
(**a**) Feature extraction heatmap of the original YOLOv5 model; (**b**) feature extraction heatmap after introducing the dense module; (**c**) feature extraction heatmap of the Res-DenseYOLO model.

During the process of image detection by the model, the alignment between the model’s focus on the image and the actual location of the target class is of great importance in evaluating the model’s performance. Through heatmaps, we can intuitively understand the areas that the model focuses on. The darker the color in the heatmap, the more concentrated the model’s feature extraction is in that area. To validate the correctness of feature extraction by different models, we selected the original YOLOv5 model, YOLOv5 with added dense network module, and our improved YOLOv5 model for feature extraction, as shown in Figure 9.

From Figure 9, we can see that the original YOLOv5 model (Figure 9a) lacks focused feature extraction capability. In the area where there was no cow in the aisle, the model still partially extracted features from the aisle region, which reduced the feature extraction performance of the network and led to lower accuracy. After adding the dense network module (Figure 9b), the detection model could better extract features from the cows to be detected. However, it still failed to pay attention to the position information of cows in the edge regions of the image. In the heatmap of our improved Res-DenseYOLO model (Figure 9c), we can observe that the color in the area where cows gathered for drinking was darker compared to the previous models, indicating a stronger feature extraction capability of the network. Additionally, the model could also focus on the positions of cows standing at the edges. Therefore, the improved model can effectively identify the areas where cows frequently appear and pay attention to them while also detecting cows in occasional areas, demonstrating the model’s robustness and accuracy.

#### 3.3.3. Comparative Analysis of Model Performance

**Figure 10:**
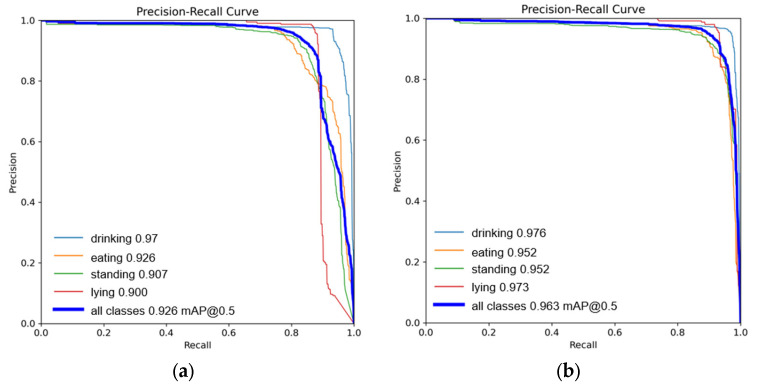
(**a**) P-R curve of the YOLOv5 model; (**b**) P-R curve of the Res-DenseYOLO model.

In this section, we compare the original YOLOv5 model with our improved Res-DenseYOLO model, as shown in Figure 10. The precision-recall curve is plotted with re-call as the x-axis and precision as the y-axis. The larger the area enclosed by the precision-recall curve, the higher the model’s baseline precision. From the graph, we can accurately observe the changes in precision and recall of the model.

From the data in the graph, we can see that the mAP (mean average precision) gap between drinking and eating behaviors is small, at 0.6% and 2.6%, respectively. However, both models have a larger mAP difference in the standing and lying behaviors, at 4.5% and 7.3%, respectively. After analyzing the images of standing and lying behaviors, we found that some cows were standing close to the end of the camera’s capture area, and some standing cow images had a dense distribution, posing a challenge for the model’s recognition of small targets. Additionally, the images of cows lying down were captured at night using infrared cameras, resulting in less prominent colors in the images and testing the model’s ability to extract image features. Therefore, the Res-DenseYOLO model, which combines dense networks and multi-head mechanisms, can effectively address the aforementioned issues. After improvement, the Res-DenseYOLO model showed a 3.7% increase in mAP compared to the original model. Thus, it can be seen that the improved Res-DenseYOLO model greatly enhanced the accuracy of cow recognition.

#### 3.3.4. Model Result Visualization Comparison and Analysis

To further validate the performance of the model in detecting different cow behaviors, we selected cow behavior images from different scenarios and used the original YOLOv5 model as our baseline model.

**Figure 11:**
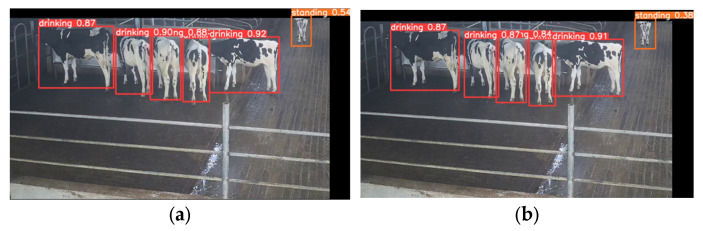
(**a**) Detection results of drinking behavior using the original YOLOv5 model; (**b**) detection results of drinking behavior using the Res-DenseYOLO model.

Figure 11 shows the drinking behavior of cows. We can observe that both detection models achieved high recognition accuracy when cows were not stacked and when they were standing in the center of the image. This is because the entire cow body was presented in the image, and the features were relatively complete, making it easier for the models to recognize them. In the case of cows with partially displayed features in the middle of the image (Figure 11b), our Res-DenseYOLO model, which incorporated the dense module to enhance feature extraction, showed a 1–3% improvement in recognition accuracy compared to the original YOLOv5 model. However, for cows standing at the edges of the image, the recognition accuracy of the model decreased due to incomplete cow features. Nevertheless, compared to the original YOLOv5 model, our model had improved accuracy by 16% and could better recognize cows with partially incomplete features that still appear in the image.

**Figure 12:**
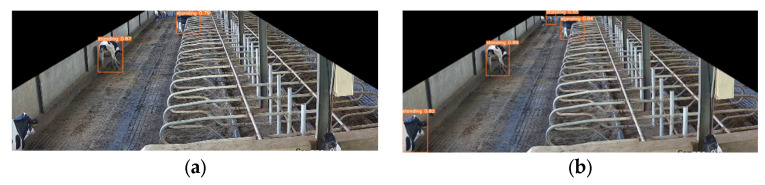
(**a**) Recognition results of the YOLOv5 original model for detecting standing behavior; (**b**) recognition results of the Res-DenseYOLO model for detecting standing behavior.

Figure 12 shows the recognition results of the model for cows standing. From the image, we can see that in the corridor of the cowshed, the size of the cow in the image varies greatly depending on its distance from the camera. Especially for cows in the far-end area of the corridor, due to their distance, their size in the image was significantly smaller, and the feature information was relatively incomplete. This presented a significant challenge for the model to recognize such small targets (Figure 12a). However, we had employed the design of multiscale detection heads to facilitate the model in better feature extraction in different regions. Additionally, the Res-Dense module enhanced the feature representation capability, allowing the model to identify target categories from incomplete features. Furthermore, the CoordAtt attention mechanism enhanced the network’s ability to expand the attention range of the network model [30].

Our Res-DenseYOLO model effectively recognized cows with standing features in far-end positions (Figure 12b), even when their size was small. Additionally, the model accurately identified cows with only the upper half of their body visible in the bottom-left corner. This fully demonstrated the effectiveness of our design strategy.

**Figure 13:**
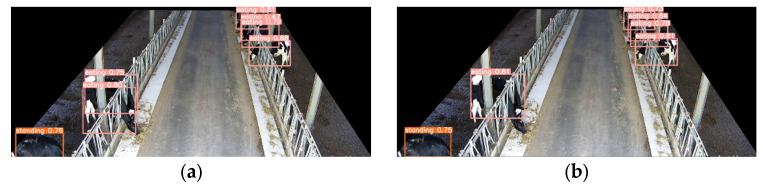
(**a**) Recognition results of the YOLOv5 original model for detecting feeding behavior; (**b**) Recognition results of the Res-DenseYOLO model for detecting feeding behavior.

Figure 13 shows the recognition results of the model for cow feeding behavior. Due to the compact design of the cow feeding trough, cows often stacked on top of each other while feeding, making it crucial for the model to accurately distinguish stacked cows. The original YOLOv5 model (Figure 13a), when dealing with such overlapping objects, can only roughly extract the overall contour features of the overlapping region, and, therefore, only one cow can be detected. However, our Res-DenseYOLO model (Figure 13b), utilizing the optimized backbone network, can deeply extract detailed features within the overlapping region. By separating the feature representation of different regions using a multi-head structure, it can accurately and clearly recognize two cows. Moreover, for cows in a feeding position on the right side, our model improved the detection accuracy by 5% compared to YOLOv5. The results confirmed the effectiveness of our enhanced design in resolving the problem of overlapping objects.

**Figure 14:**
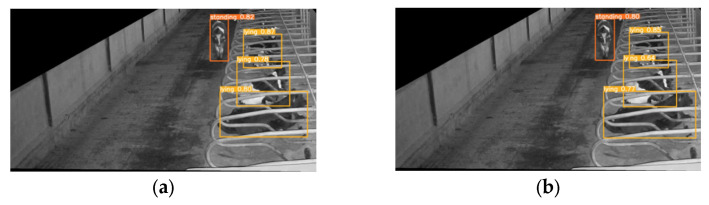
(**a**) Detection results of YOLOv5 original model for lying behavior recognition; (**b**) detection results of Res-DenseYOLO model for lying behavior recognition.

Figure 14 shows the detection results of the model for cows’ lying down behavior during nighttime. When the monitoring image exhibited reduced color contrast at nighttime, this imposed higher demands on the model’s capacity to extract discerning features from the image. However, our Res-DenseYOLO model, with the optimized backbone network, can dig deeper into the details of monochrome images. From the figure (Figure 14b), it could be observed that the Res-DenseYOLO model improved the recognition rates for both standing and lying down behavior of cows, especially when the images of two cows intersected. In such cases, Res-DenseYOLO showed a 14% increase in confidence for correctly identifying the cow lying down in the middle. The results demonstrated the robust performance of our model, even in challenging scenarios that involve intricate features.

### 3.4. Limits and Future Research

There are still some limitations in the study that cannot be ignored. Firstly, in an actual cowshed, the complexity of the environment can lead to an inability to distinguish between the cattle and the environment, and this phenomenon often occurs in areas that deviate from the camera acquisition area. The datasets collected were also mostly based on well-lit environments and did not span the winter months when temperatures are low. In the future, we will increase the time span of the data collection and enhance the data with GAN networks [31] or super-resolution techniques [32].

Second, we borrowed the idea of the DenseNet module to enhance the model’s ability to extract features. However, this approach also enhances the number of parameters of the network, which poses difficulties for deployment on mobile or embedded platforms. To solve this problem, we propose to use the following two approaches. The first one is to use model channel pruning [33] to obtain a more compact and efficient model by removing the low contributing channels when the accuracy loss is minimized. The other, in [34], proposes an extension of the Rexnet to the Rank eXpansion Network 3D algorithm (Rexnet 3D) network to achieve non-contact automatic recognition of the basic motion behaviors of cows, with a recognition accuracy of 95%. We found that in its performance comparison with other models, it obtained close to or even better accuracy with fewer FLOPs, which is worth exploring further in future research.

Finally, merely recognizing the behaviors of dairy cows does not fulfill the management requirements. Individual cow recognition will also be an important part of future research. In both [19,35], they conducted cow identity studies based on cow face patterns and back patterns, respectively, through the improved YOLO model. In future studies, we will explore the performance of the Res-DenseYOLO model on cow identity. In addition, the nose pattern of the cow can also be used as a unique identifier for its identity, to be further explored in future research work [36].

In summary, this study is dedicated to the automatic identification of cow behavior by machine vision in a real cowshed environment. The results of the experiment proved that the application of this technology is promising for monitoring the physical condition of cows and improving their well-being.

## 4. Conclusions

The objective of this research was to achieve precise identification of diverse behaviors exhibited by cows in actual dairy cowshed environments. To achieve this objective, we proposed the Res-DenseYOLO detection model based on the YOLOv5 architecture. By incorporating additional detection heads, the Res-DenseYOLO model enhanced the recognition capability of distant cow behaviors in the target image. Furthermore, we enhanced the model’s ability to extract image features in actual cowshed environments by incorporating dense modules into the existing residual modules, ensuring accuracy. In addition, the incorporation of a dense module into the original residual module enhanced the model’s capability to effectively image features, thereby ensuring its accuracy in actual dairy cowshed environments. Moreover, by incorporating the CoordAtt attention mechanism and anchor frame loss function, the convergence speed during model training was further improved. As a result of these modifications, Res-DenseYOLO achieved improvements of 0.7% in accuracy, 4.2% in recall rate, and 3.7% in mAP compared to YOLOv5. Our research model also demonstrated comparable performance advantages compared to other mainstream object detection models. The data used in this study were collected through surveillance cameras in the cowshed, eliminating the need for special acquisition equipment and providing convenience for future research on animal behavior recognition in intensive farming domains.

## Figures and Tables

**Table 1:** The environment of experiment parameters.

Configuration	Parameters
CPU	Intel(R) Xeon(R) Gold 5218R
GPU	GeForce RTX 2080 Ti
Memory	94G
Operating system	Ubuntu 16.04
Development environment	Python 3.7
Accelerated environment	CUDA 11.1

**Table 2:** Initial training parameters for the Res-DenseYOLO model.

Hyperparameters	Value
Optimization	SGD
Initial learn rate	0.01629
Momentum	0.98
Weight decay	4.5 × 10^−4^
Batch size	16
Epoch	100

**Table 3:** Performance of different backbone networks.

Backbone	Precision (%)	Recall (%)	mAP@50 (%)
ShuffleNetV2	92.3	89.3	94.2
EfficientNet	92.4	89.6	94.4
Res-DenseNet	93.1	89.9	94.8

**Table 4:** Performance of different loss functions.

Loss Function	Precision (%)	Recall (%)	mAP@50 (%)
DioU	92.1	88.1	94.3
GioU	92.2	88.6	94.8
CioU	92.2	89.0	95.1
SioU	92.5	90.2	95.3

**Table 5:** Performance of different attention mechanisms.

Method	Precision (%)	Recall (%)	mAP@50 (%)
SE	92.5	90.3	94.3
CBAM	92.9	90.2	94.6
CoordAtt	93.5	90.6	95.5

**Table 6:** Comparative results of ablation experiments. The checkmark indicates that this module has been added to the basic model.

Res-Dense	CoordAtt	SioU	4H	Precision (%)	Recall (%)	mAP@50 (%)
✓				93.1	89.9	94.8
✓	✓			93.5	90.6	95.5
✓	✓	✓		93.7	90.5	95.8
✓	✓	✓	✓	94.7	91.2	96.3

**Table 7:** Performance comparison of different models on the validation set.

Model	Precision (%)	Recall (%)	mAP@50 (%)
SSD	91.2	88.3	90.6
FAST-RCNN	90.5	87.2	87.2
YOLOV4	91.7	88.7	93.2
YOLOV5s	92.5	87	92.6
ourYOLOV5	93.5	91.2	96.3
YOLOV7-tiny	92.7	89.0	94.0

## Data Availability

The data presented in this study are available on request from the corresponding author.
